# Recurrent ST Elevation Myocardial Infarction from Norepinephrine-induced Coronary Vasospasm

**DOI:** 10.7759/cureus.7605

**Published:** 2020-04-09

**Authors:** Raed Qarajeh, Annapoorna Singh, Yevgeniy Khariton, Nikita Rafie, Paramdeep Baweja

**Affiliations:** 1 Internal Medicine, University of Missouri Kansas City School of Medicine, Kansas City, USA; 2 Cardiovascular Disease, University of Missouri Kansas City, Kansas City, USA; 3 Cardiology/Internal Medicine, Truman Medical Center, Kansas City, USA

**Keywords:** st elevation myocardial infarction, coronary vasospasm, myocardial infarction with no obstructive coronary atherosclerosis, norepinephrine

## Abstract

Myocardial infarction with no obstructive coronary atherosclerosis (MINOCA) is a distinct clinical syndrome characterized by evidence of myocardial infarction with normal or near-normal coronary arteries on angiography (stenosis severity < 50%). Coronary artery spasm, as seen in “variant angina,” usually occurs at a localized segment of an epicardial artery. Here, we present a case of a 58-year-old male who had norepinephrine-induced coronary vasospasm which resulted in ST elevation myocardial infarction on two consecutive admissions.

## Introduction

Coronary artery disease (CAD) is a major cause of death and disability in developed countries. Although CAD mortality rates worldwide have declined over the past decades, CAD remains responsible for at least one-third of all deaths in individuals over age 35 years [1]. It has been estimated that nearly one-half of middle-aged men and one-third of middle-aged women in the United States will develop some manifestation of CAD [2]. Most cases of acute myocardial infarction (MI) are caused by rupture or erosion of a fixed atherosclerotic plaque with subsequent thrombus formation. Other causes include a supply-demand mismatch in the presence of a significant fixed atherosclerotic obstruction. MI with no obstructive coronary atherosclerosis (MINOCA) is a distinct clinical syndrome characterized by evidence of MI with normal or near-normal coronary arteries on angiography (stenosis severity < 50%) [3].

## Case presentation

**Figure 1:**
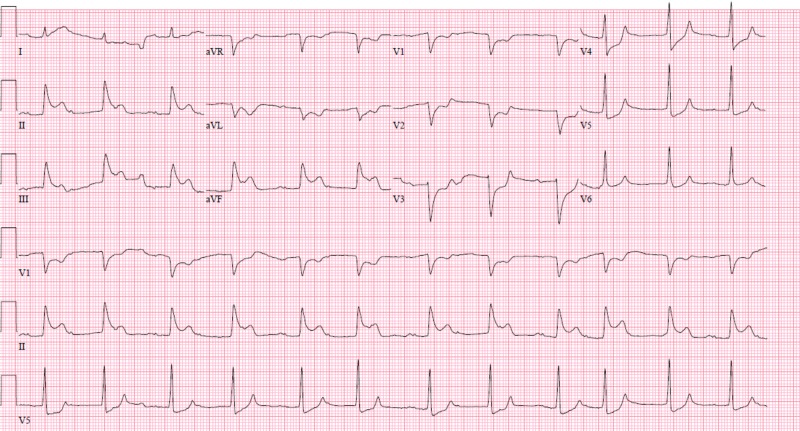
Electrocardiogram (ECG) shows ST segment elevation in the inferior leads. ECG shows atrial fibrillation, ST segment elevation in the inferior leads, and reciprocal ST segment depression.

**Figure 2:**
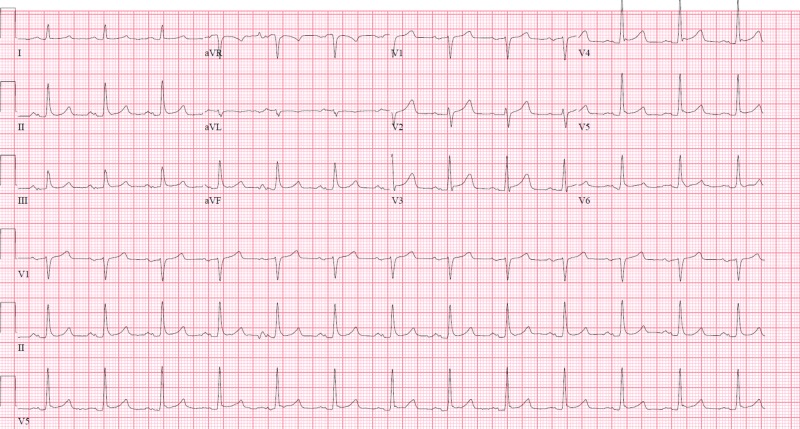
Initial electrocardiogram (ECG) on the first admission. Initial ECG on the first admission shows normal sinus rhythm without ST segment changes.

A 58-year-old male with a past medical history of hepatitis C related liver cirrhosis, small bowel obstruction status post bowel resection and ileostomy, and tobacco use presented to the hospital with several episodes of syncope for one day. These episodes were positional and occurred upon standing. The patient noted black colored stools from the ostomy, nausea, and vomiting. On presentation, he denied any chest pain or shortness of breath. On initial assessment, he was hypotensive with blood pressure (BP) of 64/42 mmHg. Fecal occult blood testing of the ostomy bag was positive, and initial hemoglobin was 12.8 g/dL. He received fluid resuscitation and was admitted to the ICU for presumed hypovolemic shock from upper gastrointestinal (GI) bleed. He remained hypotensive despite fluid resuscitation and was therefore started on low-dose norepinephrine. Esophagogastroduodenoscopy showed esophagitis and gastritis; however, there was no active upper GI bleed or esophageal varices. The following day, while the ICU team was placing an internal jugular central venous line, the patient experienced pressure-like chest pain. Electrocardiogram (ECG) at that time revealed atrial fibrillation, ST elevation in inferior leads, and ST depression in anterior leads (Figure 1) and laboratory testing was significant for elevated troponin at 0.05 ng/mL. The initial ECG on admission showed normal sinus rhythm without ST elevation (Figure 2).

**Figure 3:**
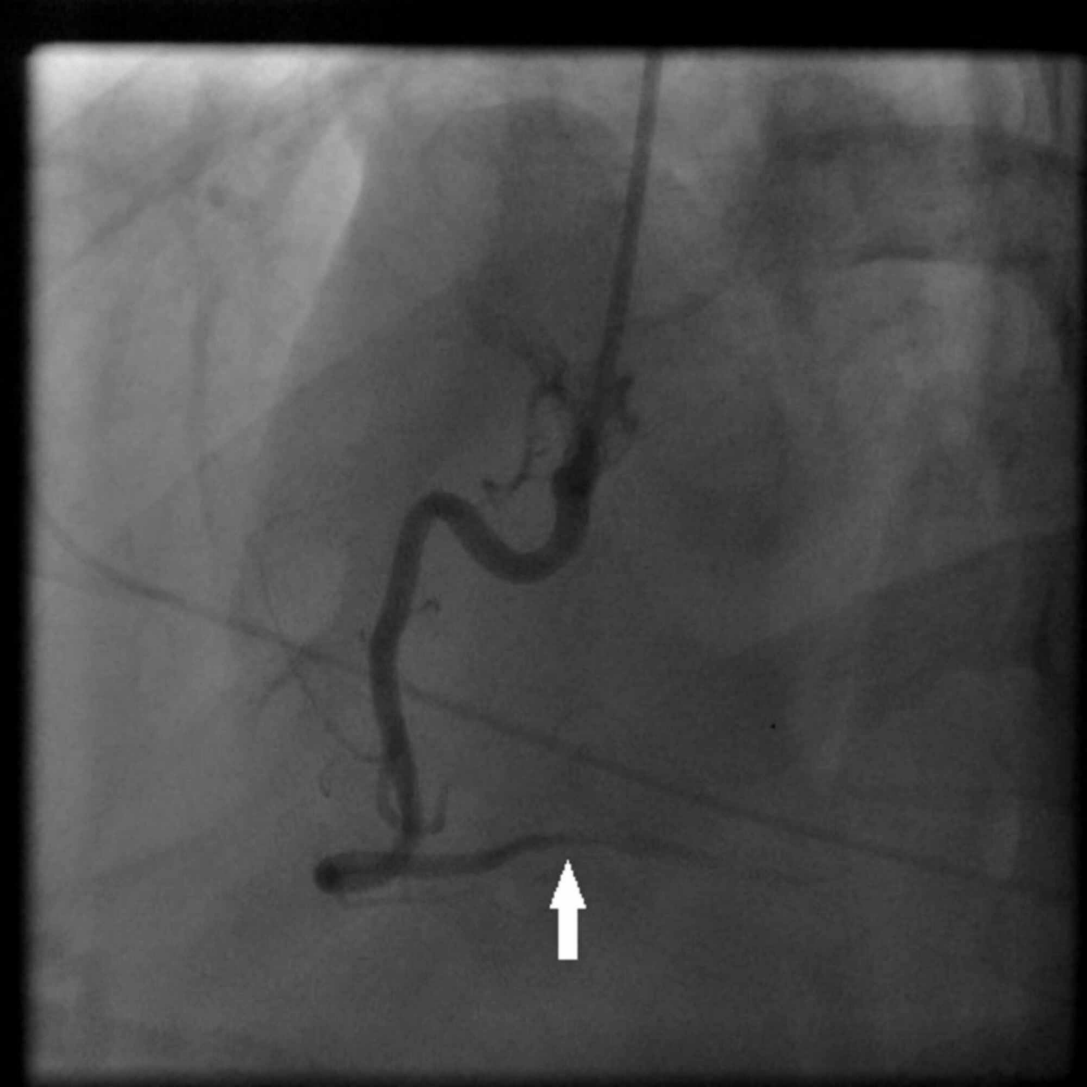
Coronary angiogram of the patient. Right coronary injection shows significant narrowing (white arrow) of the right coronary artery in the distal part.

**Figure 4:**
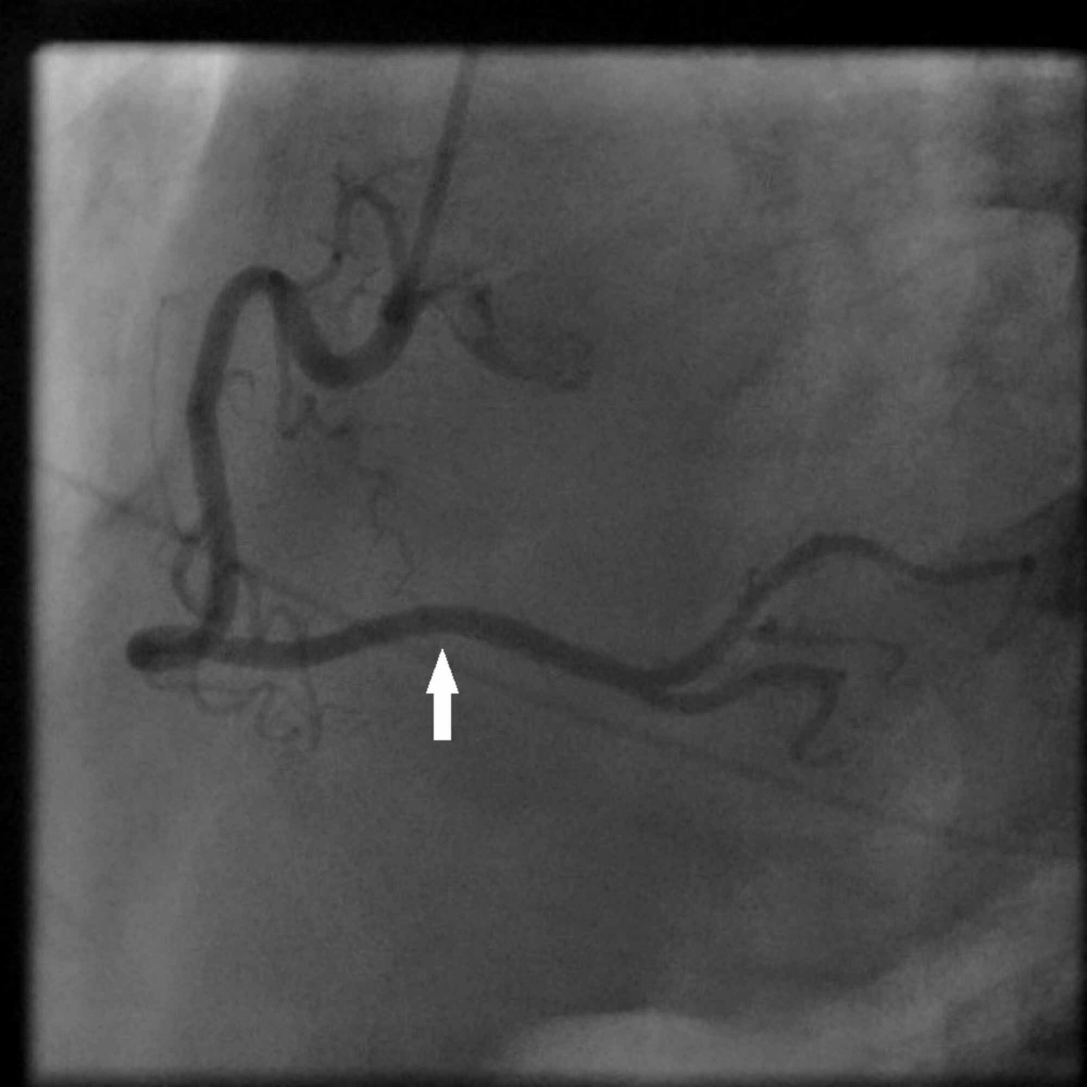
Coronary angiogram of the patient after administration of intracoronary nitroglycerin. Right coronary injection shows complete resolution of distal right coronary artery vasospasm (white arrow) after administration of intracoronary nitroglycerin. The sinoatrial nodal branch and the atrioventricular nodal artery can be seen as well.

At this time, the patient underwent urgent coronary angiography for ST elevation MI (STEMI). Interestingly, coronary angiography revealed severe vasospasm in the distal right coronary artery (RCA) that was relieved with intracoronary nitroglycerin (Figures 3, 4).

**Figure 5:**
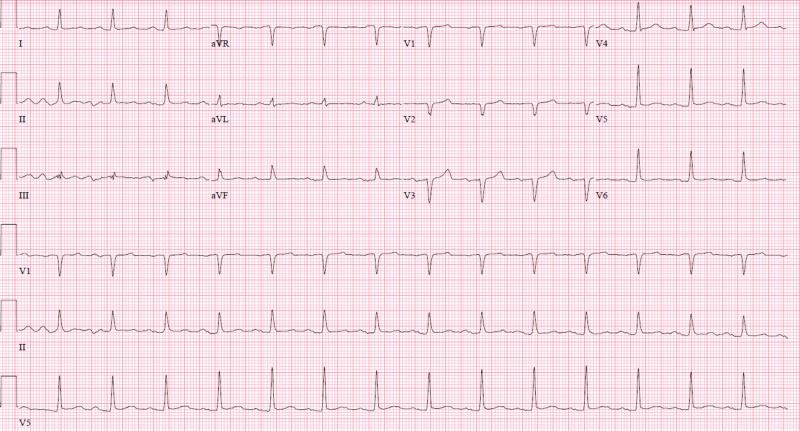
Electrocardiogram (ECG) after administration of intracoronary nitroglycerin and resolution of coronary vasospasm. ECG shows resolution of ST segment elevation.

The patient’s chest pain resolved upon resolution of vasospasm. Subsequent ECG showed normal sinus rhythm with resolution of ST elevation (Figure 5).

The patient was started on isosorbide mononitrate 30 mg daily. BP later improved and low-dose norepinephrine was stopped. He did not have any overt GI bleeding during this admission. His syncope on admission was likely due to severe hypotension of unclear etiology. The patient was discharged in a stable condition.

**Figure 6:**
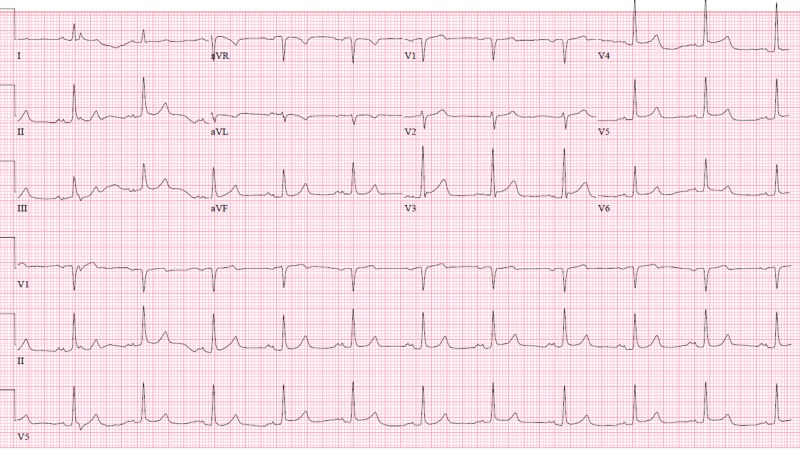
Initial electrocardiogram (ECG) on the second admission without ischemic changes. Initial ECG on the second admission shows normal sinus rhythm without ST segment changes.

Interestingly, the patient presented again, two months later, with syncope in the setting of hypotension. Initial EKG did not show any ischemic changes (Figure 6).

**Figure 7:**
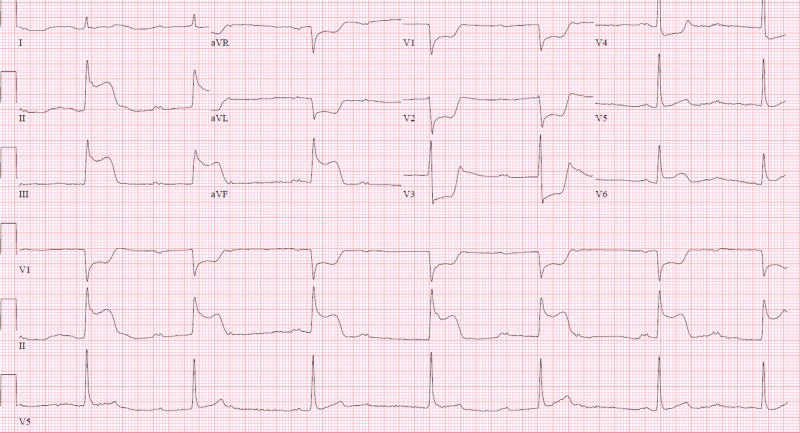
Electrocardiogram (ECG) shows ST segment elevation after administration of intravenous norepinephrine. ECG shows ST segment elevation in the inferior leads, and reciprocal ST segment depression.

He tested positive for cocaine. Mean arterial pressure was 63 mmHg despite fluid resuscitation; hence, he was started on norepinephrine. Few minutes later, the patient started complaining of severe chest pain. Repeat EKG showed inferior ST elevations with anterolateral reciprocal changes and bradycardia (Figure 7).

**Figure 8:**
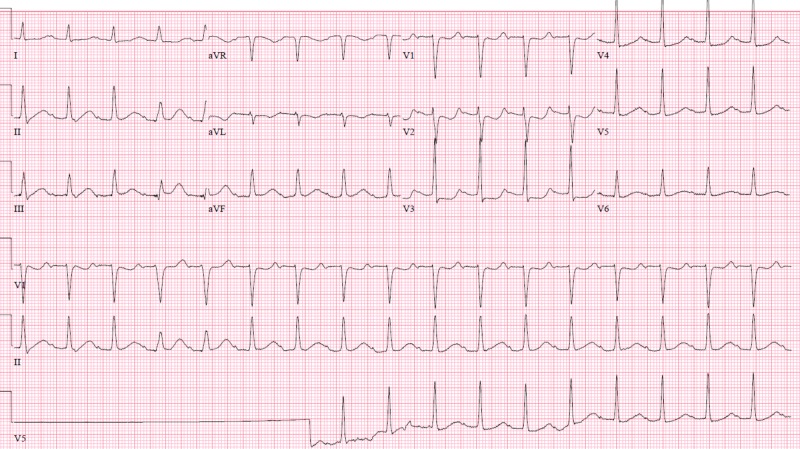
Electrocardiogram with resolution of the ST segment elevation after stopping norepinephrine and administration of nitroglycerin.

Given his previous admission and recent coronary angiogram without significant stenosis other than the RCA vasospasm, his chest pain and STEMI were thought to be again secondary to norepinephrine-induced coronary vasospasm. Norepinephrine was switched to dopamine and he was given nitroglycerine. His chest pain resolved, and serial EKG showed resolution of ST elevations with improvement in his heart rate (Figure 8).

## Discussion

MINOCA is a syndrome with many causes and may involve the epicardial vessels and/or the coronary microcirculation [3]. Coronary artery spasm, as seen in “variant angina,” usually occurs at a localized segment of an epicardial artery, but can involve two or more segments of the same (multifocal spasm) or of different (multivessel spasm) coronary arteries [4]. Among studies in patients with MINOCA, the reported prevalence of coronary artery spasm is extremely variable (3%-95%) due to the different stimuli and provocative tests used to diagnose spasm as well as the different definitions of spasm. Another variance in the prevalence of coronary artery spasm may be due to increased prevalence in certain ethnic groups, such as Asian patients compared to Caucasian patients. The Coronary Vasomotion Disorders International Study group (COVADIS) has published diagnostic criteria for vasospastic angina [5].

Vascular smooth muscle hyper-reactivity is thought to be central to the pathogenesis of vasospastic angina [6]. Spasm may occur in the absence of any preceding increase in myocardial oxygen demand (e.g., exercise), and can occur in angiographically normal coronary vessels or, more commonly, at the site of atherosclerotic plaques of variable severity. Original descriptions reported anatomically focal spasm sites, but diffuse spasm is being increasingly described [7].

Multiple vasoconstrictors have been used to provoke coronary spasm, including norepinephrine, acetylcholine, serotonin, histamine, and dopamine, suggesting that a single receptor pathway cannot explain this phenomenon [6]. Our patient developed coronary vasospasm and STEMI on two consecutive admissions after starting norepinephrine. In addition, cocaine might have contributed to coronary vasospasm in the second admission. Spasms have also been triggered by multiple common vasoconstrictors, such as ephedrine-based products, cocaine, marijuana, alcohol, butane, sumatriptan, and amphetamines [6]. Receptor antagonists (e.g., ketanserin and prazosin) do not inhibit spasm [8]. Inhibition of smooth muscle contractile mechanisms using other (non-receptor) pathways (e.g., nitrates, calcium channel blockers) is an effective means to inhibit spasm. Increased calcium sensitivity of the vascular myosin light chain, mediated by enhanced Rho kinase activity and enhanced phospholipase C activity, has been shown to also have a role [8,9]. A role for an imbalance of vagal and sympathetic tone in triggering coronary spasm has also been speculated as episodes of vasospastic angina occur more often from midnight to early morning, when vagal tone is higher [10,11]. Endothelial dysfunction may be a predisposing factor, but it is not likely the sole reason for coronary spasm given that typical coronary artery spasm is an uncommon condition, whereas endothelial dysfunction is common. Hypertension and hypercholesterolemia, two major predictors of atherosclerotic cardiovascular disease, do not accurately predict the development of vasospastic angina [11]. Cigarette smoking, however, is a major risk factor for vasospastic angina [12]. There is some evidence that genetic factors and insulin resistance are also associated with vasospastic angina [13]. Myocardial bridging, per se, is unlikely to cause MINOCA. However, it can predispose the affected artery to spasm. MINOCA patients with bridging seen on coronary angiography should be considered for further provocative testing.

Many atherosclerotic plaques expand outward rather than encroaching on the arterial lumen. These "positively remodeled” plaques are often lipid-rich and have a thin fibrous cap; they are vulnerable to rupture into the lumen [14]. Transient and partial thrombosis at the site of a non-obstructive plaque with subsequent spontaneous fibrinolysis and distal embolization may be one of the mechanisms responsible for the occurrence of MINOCA. Similarly, coronary erosion with loss of surface endothelium, possibly due to hyaluronan and neutrophil accumulation, can also cause MINOCA [14]. Intracoronary imaging and optical coherence tomography may provide additional information.

Coronary microvascular dysfunction, also referred to as cardiac syndrome X or angina pectoris with normal coronary arteries, is a process characterized by transient myocardial ischemia, as indicated by ST segment changes and angina. This may either be spontaneous or induced by intracoronary administration of acetylcholine in the absence of obstructive CAD and epicardial spasm [14]. About 25% of patients with acute coronary syndrome and no obstructive CAD have evidence of coronary microvascular dysfunction, although an elevation of troponin is infrequent [15].

It has been shown that the autonomic nervous system plays a crucial role in coronary vasospasm [16]. Both sympathetic and parasympathetic stimuli may trigger coronary vasospasm [10,17]. To our knowledge, there is only one reported case of coronary vasospasm from a painful stimulus [18]. In the first admission, our patient developed chest pain and coronary vasospasm in the setting of pain from central line placement. Pain from central line insertion might have induced vagal activation. Both sympathetic and parasympathetic stimuli, in addition to being on a vasopressor, might have had a contributing effect on coronary vasomotor tone leading to coronary artery vasospasm. Treatment of coronary artery spasm with calcium channel blocker and/or nitrates is usually sufficient, and patients have an excellent prognosis [19]. However, persistent angina can be a challenging problem in these patients. Our patient was started on isosorbide mononitrate 30 mg daily and did very well on follow-up.

## Conclusions

Coronary artery spasm usually occurs at a localized segment of a normal or diseased coronary artery. Vascular smooth muscle hyper-reactivity is thought to be central to the pathogenesis of vasospastic angina. Multiple vasoconstrictors including norepinephrine can provoke coronary spasm resulting in MINOCA. The autonomic nervous system plays a crucial role in coronary vasospasm including both the sympathetic and parasympathetic divisions. The concept of pain-induced coronary vasospasm has been described previously; however, it is rare and has only been reported in one prior case. Patients with coronary artery spasm can be treated with calcium channel blockers and/or nitrates and they have an excellent prognosis.
